# Technology-facilitated abuse as a risk factor for STI and pregnancy in early adulthood: evidence from latine adolescents in California

**DOI:** 10.1186/s12978-025-02128-5

**Published:** 2025-09-30

**Authors:** Miriam Hartmann, Marie Stoner, Carmen Guadalupe Ramirez, Erica N. Browne, Alondra Jaime-Aguilar, Danielle Rodriguez, Nivedita L. Bhushan, Mary Kate Shapley-Quinn, Marissa Raymond-Flesch, Alexandra Minnis

**Affiliations:** 1 Women’s Global Health Imperative, RTI International, Berkeley, CA USA; 2 Department of Global Public Health, Karolinska Institutet, Stockholm, Sweden; 3 Center for Communication and Engagement Research, RTI International, Research Triangle Park, NC USA; 4 Division of Adolescent and Young Adult Medicine, Department of Pediatrics, University of California, San Francisco, CA USA; 5 Institute for Health Policy Studies, University of California, San Francisco, CA USA

**Keywords:** Technology-facilitated abuse, Adolescents and young adults, Sexual and reproductive health

## Abstract

**Background:**

Background Technology-facilitated abuse (TFA) has emerged as a significant form of violence against adolescents and young adults (AYA). However, evidence is limited regarding the prevalence of forms of TFA among AYA and how TFA from partners influences other outcomes related to AYA sexual health and wellbeing. The aim of this study was to examine the prevalence of TFA exposure among AYA in an agricultural region in the United States, to identify associated risk factors, and assess its relationship to later sexual, mental, and violence-related health outcomes.

**Methods:**

We analyzed data from a prospective cohort study of eighth graders from Salinas, California followed over eight years. Pregnancy and sexually transmitted infection (STI) testing were conducted in emerging adulthood (~age 20). TFA was measured using a 6-item scale of Cyber Dating. Log-binomial models were used to estimate risk ratios (RR) and 95% confidence intervals (CIs) for the associations between any TFA in early and middle adolescence (~ages 13-15) with sexual health, mental health and violence outcomes in emerging adulthood.

**Results:**

Among 373 participants with follow-up data, the median age of participants at baseline was 13.7 years (interquartile range (IQR) 13.4, 14) and the majority were female (56.0%, n=216), Latine (95.9%, n=370) and had at least one parent or grandparent from Mexico (88.9%, n=343). Over the entire study period in early adolescence, 41.7% (n=161) of participants reported ever having TFA experiences but the percentage was roughly 20% at any one visit. The most reported TFA type was a partner repeatedly contacting the participant via some form of technology to see where they were/who they were with. Exposure to TFA in early or middle adolescence was associated with a higher likelihood of pregnancy before age 20 (RR 1.71; 95% CI 1.02, 2. 84) and an STI diagnosis in emerging adulthood (RR 2.22; 95% CI 1.19, 4.16) in adjusted models.

**Conclusions:**

TFA was relatively common among AYA throughout adolescence and into emerging adulthood. TFA was associated with teen pregnancy and STI acquisition. Further work is needed to understand mechanisms for this relationship and to integrate TFA into existing intimate partner violence prevention programming and reduce the negative effects of TFA on sexual and reproductive health.

**Plain English summary [250 words max]:**

Technology is increasingly being used as a tool for abuse in relationships, especially among teenagers and young adults. This study looked at how common technology-facilitated abuse (TFA) is among adolescents and young adults in an agricultural region of California. We also explored how experiencing this type of abuse in early and middle adolescence (around ages 13-15) might affect reproductive health outcomes later in early adulthood.We followed a group of young people from their early teenage years into adulthood, checking in with them over eight years. At around age 20, we also tested for pregnancy and sexually transmitted infections (STIs). TFA was measured by asking whether a romantic partner had used technology, such as texting or social media, to control or monitor them.We found that about 1 in 5 participants had experienced TFA during early adolescence, and this pattern remained consistent over time. The most common form of TFA was a partner repeatedly messaging or calling to check their location and who they were with. Our findings suggest that experiencing TFA as a teenager was linked to a higher chance of pregnancy before age 20 and a greater likelihood of being diagnosed with an STI in young adulthood.These results highlight that TFA is a serious and common issue that may have long-term impacts on young people’s health. More research is needed to understand why this link exists and to develop ways to prevent TFA and support young people in safe and healthy relationships.

**Supplementary Information:**

The online version contains supplementary material available at 10.1186/s12978-025-02128-5.

## Background

Intimate partner violence (IPV) is common in the United States (U.S.) and prevention constitutes an important public health priority due to adverse consequences across multiple health and wellbeing outcomes over the life course [1]. IPV starts early with 1 in 4 women and 1 in 5 men first experiencing it before age 18 [2]. The rapid expansion of digital technology has introduced new forms and modes to perpetuate IPV, which disproportionately affects young people. Technology-facilitated abuse (TFA), defined as the use of digital tools to harass, control, or threaten individuals, is an example of this, and includes a range of behaviors, from cyberbullying to intimate partner surveillance [3–5]. While evidence of the effects of TFA are still limited, emerging data suggests that like other forms of IPV, it impacts mental health, self-efficacy to enact preventative health behaviors, and health service utilization, and may ultimately extend to sexual and reproductive health outcomes (SRH) [6, 7]. 

Given high proportions of Latine adolescents report constantly being online [8] and face disproportionately high rates of unintended pregnancy and sexually transmitted infections (STIs), examining their TFA exposure and its potential role in SRH outcomes constitutes a research priority. The concern for TFA’s impact on SRH is particularly pertinent for Latine youth in California, who account for 77% of all teen births in the state with higher rates among rural youth compared to urban peers [9, 10]. Among Latine youth, especially those in rural areas, additional barriers to care such as limited accessibility of culturally sensitive health services, other intersecting structural vulnerabilities, and experience of stigma and discrimination, may compound challenges to adopting health-promoting behaviors, leading to disproportionately adverse reproductive health effects [11, 12]. ^,^ [13] Yet, evidence is limited on the prevalence and characteristics associated with TFA among Latine youth and very few studies have used longitudinal data to examine how TFA may influence SRH outcomes among youth over time. Rigorous longitudinal evidence is needed to better identify those most at risk for TFA, inform the development of interventions to better prevent TFA, and address the consequences of TFA among youth.

The aim of this study was therefore to examine the prevalence of TFA exposure over time among Latine youth between early adolescence and young adulthood, to identify associated risk factors, and assess its relationship to SRH outcomes. By focusing on this understudied area, we aim to inform SRH research and interventions that address the unique needs of Latine youth exposed to TFA.

## Methods

### Study design

**Fig. 1:**
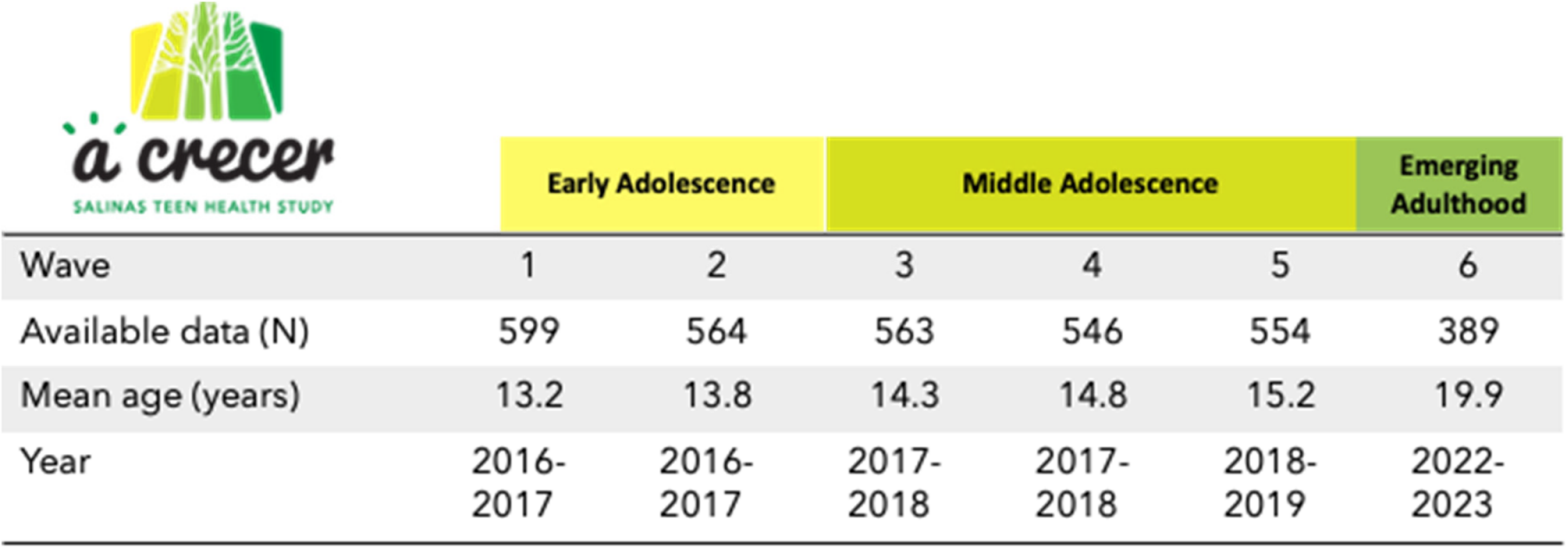
Study Design

We used data from the prospective cohort study *A Crecer*, which aims to explore social factors influencing sexual health outcomes throughout adolescence and into emerging adulthood. The study followed a community-based participatory research (CBPR) approach and was thus developed in partnership with the Monterey County Health Department and with input from youth and community advisory boards, who informed our research questions [14]. CBPR has been demonstrated to be of particular importance when conducting research with vulnerable populations, including youth and migrants, and when conducting research on sensitive topics such as violence and SRH [13]. It follows eighth graders from four public middle schools in Salinas, California. Here we present data collected from 2015 to 2023. Participants had a total of six study visits, referred to as waves 1–6, between the ages of 13 to 20 (Fig. 1). A description of the cohort and more detail about the CBPR approach are available elsewhere [14, 15].

### Setting

Salinas is an agricultural hub which produces much of the nation’s lettuce and other crops. While it benefits from a rich cultural diversity and strong community ties, it also faces challenges such as high poverty rates and community violence, which may negatively impact adolescent health. The area’s proximity to fresh produce and outdoor spaces provides opportunities for healthy living, but socioeconomic disparities prevent access to these opportunities and limited access to healthcare also present ongoing health concerns for youth.

### Participants

Eligible participants were aged 12 to 15 years old, able to complete the study procedures in English or Spanish, intended to live in Salinas for the next year, and were willing to provide contact information for a parent. Of the 599 participants who were enrolled in the original cohort (Wave 1–5), 389 (65%) participants re-enrolled at Wave 6 approximately 6 years after initial enrollment (wave 6 mean age: 20 years). Our analysis includes all participants who reenrolled at Wave 6 to examine TFA exposures in early and middle adolescence (Waves 1–5, ages 13–15) with health outcomes in emerging adulthood (Wave 6).

### Data collection

Surveys were interviewer-administered approximately every 6-months during Waves 1–5 and again at Wave 6 using computer-based questionnaires in English and Spanish (Fig. 1). Sensitive questions were completed independently by participants using audio computer-assisted self-interviewing (ACASI) in early and middle adolescence (Waves 1–5), and CASI without audio in emerging adulthood (Wave 6). Surveys captured data on structural (e.g. community-level protective and risk factors), interpersonal (e.g. family, peer, and partner relationship factors), and individual level (e.g. substance use, relationship qualities, mental health) factors theorized to influence SRH outcomes (e.g. self-reported sexual behaviors, partner violence, and health care service access). All Wave 1–5 study visits in early and middle adolescence were conducted in-person. In emerging adulthood, Wave 6 interviews were done in person or with a study interviewer over the phone or via videoconference. To facilitate accessibility of study participation, asynchronous visits could also occur with participants completing study interviews independently on a computer or smartphone. Data collection in emerging adulthood also included biological testing for chlamydia and gonorrhea (via vaginal swab or urine) and pregnancy (via urine sample). Participants who completed their visit remotely were offered a self-sample collection kit for STIs and pregnancy, including instructions for sample collection, which they returned by prepaid mail for laboratory analysis. Participants also had the option to upload STI results if they recently tested with an external medical provider within the same month of their visit or if they planned to test with an external medical provider after their visit.

### Measures

The outcomes that were examined at Wave 6 in emerging adulthood were related to SRH, mental health (i.e. symptoms of anxiety and symptoms of depression) and violence (IPV victimization, IPV perpetration, TFA). Symptoms of depression (yes/no) were measured using the Patient Health Questionnaire-8 (PHQ-8) for adolescents using a cutoff of ≥ 10. The measure has been validated as correlated with the diagnosis of depression in national samples and Latine populations and used in prior publications from the A Crecer study [16, 17]. Symptoms of anxiety were measured using the General Anxiety Disorder 7-item scale (GAD-7), which is widely used to screen for GAD, assessing frequency of experiences over the past 2 weeks with scores ranging from 0 to 21 (0–9 no to mild anxiety; 10–14 moderate; 15–21 severe) [18]. In-person IPV victimization (yes/no) and IPV perpetration (yes/no) were asked in relation to the past six months, and measured using a five-item version of the Revised Conflict Tactics Scale [19–21]. Items included experiencing from a partner (victimization) or doing any of the following to a partner (perpetration): calling them names, insulting them, or treating them disrespectfully in front of others; swearing at them; threatening with violence or to hurt them; pushing or shoving them; or throwing something at them that could hurt them. The latter two items were considered physical abuse and the remaining items, psychological abuse.

SRH outcomes included teen pregnancy, any STI diagnosis, visited a clinic for sexual health services in the last 12 months (yes/no), participant or their partner used any birth control method other than condoms to avoid pregnancy in the last 12 months (always, sometimes, never), condom use at last sex (yes/no), and use of emergency contraception in the last 12 months (yes/no). Teen pregnancy was defined as becoming pregnant or getting someone pregnant before age 20. Participants assigned female sex at birth were asked if they had ever been pregnant and how old they were when their first pregnancy began. The self-reported teen pregnancy measure was complemented by urine-based pregnancy testing at the visit in emerging adulthood. Participants assigned male at birth were asked if they had ever gotten someone pregnant and how old they were when they first got someone pregnant.

Diagnosis of any STI was measured using a combined measure of self-report and biologically-detected chlamydia and/or gonorrhea. Via self-report, all participants were asked “Have you ever been told by a doctor, nurse or other health care provider that you had a sexually transmitted infection?” If they answered yes, they were asked to select which STI had been diagnosed. Additionally, chlamydia and gonorrhea testing was conducted using a nucleic acid amplification test from a first catch urine sample or self-obtained vaginal swab (for participants with a vagina). Those testing positive were notified of their results by a study physician and referred for treatment to a provider or clinic of their choice at no cost to participants.

The exposure of TFA was self-reported using a 6-item scale of Cyber Dating Abuse that has been previously validated and used in AYA [22]. Responses were on a five-point Likert scale of frequency ranging from never to every day. Participants were asked “*In the past 6 months*,* how many times has a partner done any of the following things using mobile apps*,* social networks*,* texts or other digital communication*?” At wave 6, they were asked in relation to the past 12 months. Example items included “made mean or hurtful comments to you,” “repeatedly contacted you to see where you were/who you were with,” and “spread rumors about you.” Participants were defined as having experienced any TFA if responded that they were repeatedly contacted by a partner to see where they were/who they were with every day or almost every day or if they had ever experienced any of the other items. Participants who did not have a partner were coded as not having experienced TFA.

Drawing from approaches used in other violence exposure scales [23, 24], which incorporate frequency and severity into their assessment, an intensity score was also created from the TFA items. This was done by multiplying the number of abusive behaviors experienced by the frequency that they were experienced and by a measure of severity for each item. Severity scores ranged from 1 to 3 where 1 = mild, 2 = moderate, 3 = severe. Items were scored by a group of staff (MH and MS and reviewed by the team) and were based on their prevalence, and assessments of the psychological or physical harm they are likely to cause. For example, posting nude pictures or image-based abuse, which was less common and has been associated with severe mental health concerns including PTSD and suicidality [25], was ranked higher than mean comments. The TFA intensity score was further categorized into low [1–3], medium [4–10], and high (10 and up) scores based on the distribution of scores within the sample.

### Covariates

Potential confounders were measured at baseline and included age (years), sex at birth, neighborhood disorder (high vs. low), mother’s educational level (less than high school, high school/GED, more than high school), food insecurity (yes/no), and symptoms of depression (yes/no). Neighborhood safety was measured by the number of disorder events exposed to, out of 11 types, during the past year (e.g., “I saw people dealing drugs near my home”). A sum of the total number of events occurring at least once during the past year was calculated and was dichotomized as high (≥ 5 events) versus low (< 5 events) based on prior analyses [15]. Any substance use was defined as self-reporting use of alcohol, marijuana, prescription drugs or illicit drugs in the past 6 months. Food insecurity, was measured with two questions that assess limited or uncertain access to adequate food aligned with USDA assessments (e.g., “In the last 6 months, were you ever hungry but didn’t eat because there wasn’t enough money for food?” and “In the last 6 months did you ever eat less than you felt you should because there wasn’t enough money to buy food?”) [26]. 

### Data analysis

First, we examined baseline (Wave 1) demographic characteristics among those who did and did not experience TFA at the Wave 2 study visit when it was first asked (median age 13.8 years). We reported frequencies and percentages for categorical variables and medians with the interquartile range (IQR) for continuous variables. We then described individual TFA items over time at all visits and bivariate relationships between experiences of any TFA in early and middle adolescence (~ ages 13–15; wave 2–5) with outcomes in emerging adulthood (~ age 20; wave 6). Again, we reported frequencies and percentages for categorical variables and medians with the interquartile range (IQR) for continuous variables. Next, we used log-binomial models to estimate risk ratios (RR) and 95% confidence intervals (CIs) for the associations between any TFA experienced from partners in early and middle adolescence (wave 2–5) with sexual health, mental health and violence outcomes in emerging adulthood (wave 6). Log-binomial models were selected because the outcome was dichotomous and because odds ratios are inflated if the outcome is common. In the case that log-binomial models did not converge, we used log-Poisson models with robust standard errors. We also explored interactions between TFA and sex at birth in all models and examined associations with TFA intensity and outcomes using log-binomial models similar to those described above.

### Ethics

At study enrollment in 8th grade (Wave 1), all adolescents provided informed assent to participate following verbal parent permission. At the visit in emerging adulthood (Wave 6), all participants provided written informed consent as they were aged 18 years or older. Incentives were provided at each study visit and all youth received a referral guide to relevant community resources. At Waves 1–5, participants who screened positive for depression or had other mental health needs were offered a fast-track referral to behavioral health services through a mechanism established by the study with the local health department. At Wave 6, participants testing positive for STIs were contacted by a study physician to ensure treatment was accessed and available free of cost. Participants who reported experience of violence or screened positive for depression or had other mental health needs were offered referrals to behavioral health services. The RTI International Institutional Review board reviewed and approved all study procedures.

## Results

### Participant characteristics

A total of 386 participants had TFA data available on any TFA from wave 2–5 and were included in this analysis. The median age at enrollment was 13.7 years (interquartile range (IQR) 13.4, 14; Table 1). The majority of participants were female (56.0%, *n* = 216), Latine (95.9%, *n* = 370), of Mexican heritage (88.9%, *n* = 343), and were US-born to immigrant parent(s) (72.0%, *n* = 278). Around half (50.0%, *n* = 193) of participants had at least one parent who works in agriculture. At baseline (Wave 1), approximately 14% of the population reported symptoms of depression (13.7%, *n* = 53) and had used any alcohol, marijuana, prescription drugs, or illicit drugs in the past 6 months (17.4%, *n* = 67). Most (73.8%, *n* = 285) participants lived with both parents, 99.7% (*n* = 385) had contact with their mother, 90.2% (*n* = 348) had contact with their father, and 8.3% experienced food insecurity in the past 6 months (*n* = 32).

**Table 1:** Baseline sociodemographic and psychosocial characteristics in early adolescence (wave 1) by experience of any technology facilitated abuse (TFA) from Wave 2[AM1] [MS2] to 5 in early and middle adolescence [AM1]Perhaps a discussion point among us: the outcome in later tables is TFA experienced at all over time in Phase 1 - do you think it should be the same here (as in T2)? [MS2]Redid this for TFA from Wave 2-5 at any visit

	**Any TFA**						
	**No (n=225, 58.3%)**		**Yes (n=161, 41.7%)**		**Total (n=386)**		**p-value**
	**No.**	**%**	**No.**	**%**	**No.**	**%**	
Age, median (IQR)	13.7	13.4, 14	13.7	13.4, 14	13.7	13.4, 14	0.701
Sex							
Male	100	44.4	70	43.5	170	44.0	0.850
Female	125	55.6	91	56.5	216	56.0	
Latine/-x ethnicity (at least one parent/grandparent)	211	93.8	159	98.8	370	95.9	0.016
Immigrant Generation							
1st: born outside the US	26	11.6	18	11.2	44	11.4	0.826
2nd: US born/immigrant parent(s)	161	71.6	117	72.7	278	72.0	
3rd+: US born and parents US born	36	16.0	23	14.3	59	15.	
US born, generation unknown	2	0.9	3	1.9	5	1.3	
At least one parent/grandparent from Mexico	197	87.6	146	90.7	343	88.9	0.336
At least one parent works in agriculture	108	48.0	85	52.8	193	50.0	0.353
Crowded housing conditions							
No	101	44.9	85	52.8	186	48.2	0.068
Crowding	100	44.4	53	32.9	153	39.6	
Severe Crowding	24	10.7	23	14.3	47	12.2	
Food insecurity/hunger, past 6 months	11	4.9	21	13.0	32	8.3	0.004
Frequent lifetime moves (5 or more)^a^	33	14.7	35	21.7	68	17.6	0.187
Neighborhood environment safety: Neighborhood disorder events experienced past 12 months, 8th grade							
<5 types of events	90	40.0	44	27.3	134	34.7	0.010
5+ types of events	135	60.0	117	72.7	252	65.3	
Living situation							
Both parents (at least part time)	173	76.9	112	69.6	285	73.8	0.212
Mother only	46	20.4	41	25.5	87	22.5	
Other	6	2.7	8	5.0	14	3.6	
Has contact with mother	225	100.0	160	99.4	385	99.7	0.237
Has contact with father	204	90.7	144	89.4	348	90.2	0.690
Mother's education							
Less than high school	105	46.7	66	41.0	171	44.3	0.325
High school/GED	55	24.4	53	32.9	108	28.0	
More than high school	60	26.7	38	23.6	98	25.4	
(Unknown)	5	2.2	4	2.5	9	2.3	
Symptoms of depression (PHQ-8-A)	23	10.2	30	18.6	53	13.7	0.018
Substance use in the past 6 months (alcohol, weed, illicit, prescription)	21	9.3	46	28.7	67	17.4	<0.001

7 participants were missing responses to number of moves

*IQR* Interquartile range, *TFA* Technology-facilitated abuse and included 7 items measuring exposure to abusive behaviors from a partner occurring online or over the phone, *PHQ-8-A* Patient Health Questionnaire-8 tailored for adolescents

^a^ lifetime moves reported at visit 2

### Exposure to TFA

A total of 161 (41.7%) reported TFA at any visit between wave 2–5. Most (21.2% *n* = 82) experienced TFA at 1 visit only, 10.4% (*n* = 40) at 2 visits, 7.8% (*n* = 30) at 3 visits, and 2.3% (*n* = 9) at 4 visits. At the first visit that these questions were asked (Wave 2), 20.9% (*n* = 80) of participants reported TFA experience in the past 6 months (Table 2), The most reported behavior experienced was a partner repeatedly contacting the participant to see where they were/who they were with. More than a quarter of participants reported this occurring at least a few times (26.2%, *n* = 79) with approximately 8% reporting the occurrence of this behavior every day or almost every day (7.6%, *n* = 23). The next most common behavior to experience was a partner making mean or hurtful comments over technology with 15.5% (*n* = 47) of participants reporting that this occurred at least a few times in the last 6 months. The remaining items were reported by a range of approximately 1–10% saying they had occurred a few times or more frequently. Reporting of individual TFA items were also similar by sex at birth (Supplementary Table 1) and remained similar throughout adolescence into emerging adulthood with approximately 20% of participants having experienced any TFA at each visit (Table 2). TFA was similar for those who did and did not re-enroll in wave 6 of the study (41.7% vs. 47.5% *p* = 0.198).

**Table 2:** Experiences of TFA over time during the study

** Wave**																										
	**Early and middle adolescence (~ages 13-15)**																				**Emerging Adulthood (~age 20)**					
	**Wave 2 (n=558)**			**Wave 3 (n=561)**						**Wave 4 (n=543)**					**Wave 5 (n=551)**						**Wave 6 (n=389)**					
	**No.**	**%**		**No.**			**%**			**No.**		**%**			**No.**		**%**				**No.**		**%**			
Partner repeatedly contacted you to see where you were/who you were with																										
Never	85	28.9		75			25.6		105			34.8		105			33.4			171			56.6			
A few times	105	35.7		110			37.5		101			33.4		126			40.1			79			26.2			
Once or twice a month	25	8.5		29			9.9		26			8.6		30			9.6			14			4.6			
Once or twice a week	27	9.2		29			9.9		24			7.9		22			7.0			15			5.0			
Every day or almost every day	52	17.7		50			17.1		46			15.2		31			9.9			23			7.6			
Partner made mean or hurtful comments to you																										
Never	244	82.7		254			86.7		270			89.4		266			84.7		256				84.5			
A few times	44	14.9		32			10.9		26			8.6		36			11.5		36				11.9			
Once or twice a month	3	1.0		4			1.4		2			0.7		9			2.9		4				1.3			
Once or twice a week	1	0.3		1			0.3		4			1.3		3			1.0		2				0.7			
Every day or almost every day	3	1.0		2			0.7		0			0.0		0			0.0		5				1.7			
Partner spread rumors about you																										
Never	259	87.8		268			91.5		280			92.7		290			92.4		283				94.6			
A few times	30	10.2		19			6.5		19			6.3		21			6.7		11				3.7			
Once or twice a month	3	1.0		3			1.0		3			1.0		1			0.3		2				0.7			
Once or twice a week	1	0.3		0			0.0		0			0.0		0			0.0		1				0.3			
Every day or almost every day	2	0.7		3			1.0		0			0.0		2			0.6		2				0.7			
Partner made a threatening or aggressive comment to you																										
Never	279	94.9		277			94.5		292			96.7		295			93.9		287				95.0			
A few times	12	4.1		11			3.8		8			2.6		12			3.8		8				2.6			
Once or twice a month	0	0.0		1			0.3		2			0.7		3			1.0		2				0.7			
Once or twice a week	1	0.3		3			1.0		0			0.0		4			1.3		2				0.7			
Every day or almost every day	2	0.7		1			0.3		0			0.0		0			0.0		3				1.0			
Partner tried to get you to talk about sex when you did not want to																										
Never	257	87.1		254			86.7		272			90.1		275			87.6		270				89.7			
A few times	27	9.2		25			8.5		25			8.3		30			9.6		18				6.0			
Once or twice a month	4	1.4		4			1.4		3			1.0		5			1.6		5				1.7			
Once or twice a week	3	1.0		2			0.7		1			0.3		2			0.6		4				1.3			
Every day or almost every day	4	1.4		8			2.7		1			0.3		2			0.6		4				1.3			
Partner asked you to do something sexual that you did not want to do																										
Never	257	87.4		254			86.7		271			89.7		280			89.2		273				90.4			
A few times	28	9.5		27			9.2		22			7.3		27			8.6		13				4.3			
Once or twice a month	2	0.7		2			0.7		4			1.3		2			0.6		7				2.3			
Once or twice a week	2	0.7		3			1.0		4			1.3		4			1.3		4				1.3			
Every day or almost every day	5	1.7		7			2.4		1			0.3		1			0.3		5				1.7			
Partner posted or publicly shared a nude or seminude picture of you																										
Never	283	96.3		286			97.6		291			96.4		303			96.5		299				98.7			
A few times	9	3.1		5			1.7		7			2.3		10			3.2		1				0.3			
Once or twice a month	0	0.0		0			0.0		3			1.0		0			0.0		1				0.3			
Once or twice a week	0	0.0		1			0.3		1			0.3		0			0.0		0				0			
Every day or almost every day	2	0.7		1			0.3		0			0.0		1			0.3		2				0.7			
Any TFA^a^	124	22.2		113			20.1		102			18.8		99			18.0		80				21.0			

TFA=technology facilitated abuse

^a^Participants were defined as having experienced any TFA if responded that they were repeatedly contacted by a partner to see where they were/who they were with every day or almost every day or if they had ever experienced any of the other items

Participants who had reported any TFA from waves 2–5 were more likely at enrollment (Wave 1) to be Latine (98.8% vs. 93.8%, *p* = 0.016), food insecure (13.0% vs. 4.9%, *p* = 0.004), report lower neighborhood safety (72.7% vs. 60.0%, *p* = 0.010), experience symptoms of depression (18.6 vs. 10.2%, *p* = 0.018) and have ever used substances (28.7% vs. 9.3% *p* < 0.001; Table 1). Experiences of any TFA from Wave 2–5 were similar by sex at birth (43.5% among males vs. 56.5% among females; *p* = 0.850).

### Associations between TFA and SRH outcomes

Table 3 shows bivariate relationships between any experience of TFA in early and middle adolescence (Wave 2–5) with outcomes in emerging adulthood (Wave 6). Participants who had experienced any TFA were more likely to have a teen pregnancy (< 20 years; 20.5% vs. 10.5%, *p* = 0.007), have any STI diagnosis through self-report or testing (17.3% vs. 6.7%, *p* = 0.001), to have visited a clinic for sexual health services in the last 12 months (39.2% vs. 26.5%, *p* = 0.008), and to have been a victim of IPV (19.0% vs. 11.6%, *p* = 0.045). Any experience of TFA in early or middle adolescence (wave 2–5) did not have a statistically significant association (*p* < 0.05) with condom use at last sex, depression, anxiety, IPV perpetration or TFA in emerging adulthood (wave 6).

**Table 3:** Outcomes in emerging adulthood (wave 6) by any TFA experienced from partners during early and middle adolescence (wave 1–5)

	Any TFA						
	No (*n* = 225, 58.3%)		Yes (*n* = 161, 41.7%)		Total (*n* = 386)		*p*-value
	No.	%	No.	%	No.	%	
Teen pregnancy (pregnancy < age 20)	**23**	**10.5**	**32**	**20.5**	**55**	**14.6**	**0.007**
Visited clinic for sexual health services in last 12 months	**59**	**26.5**	**62**	**39.2**	**121**	**31.8**	**0.008**
Any STI diagnosis	**15**	**6.7**	**27**	**17.3**	**42**	**11.1**	**0.001**
Frequency of use of birth control							
Always	39	30.0	29	23.4	68	26.8	0.327
Sometimes	39	30.0	47	37.9	86	33.9	
Never	52	40.0	48	38.7	100	39.4	
Emergency contraception use in last 12 months	78	60.0	78	62.4	156	61.2	0.694
Condom use at last sex	49	38.3	57	45.6	106	41.9	0.238
Symptoms of depression	41	18.6	34	22.8	75	20.3	0.317
Symptoms of anxiety	135	60.0	111	68.9	246	63.7	0.072
IPV Victimization yes/no	**26**	**11.6**	**30**	**19.0**	**56**	**14.7**	**0.045**
IPV Perpetration yes/no	27	12.1	26	16.5	53	13.9	0.220
Digital IPV Victimization yes/no	41	18.3	39	24.8	80	21.0	0.123

*IPV* Intimate partner violence, *TFA* Technology facilitated abuse

Experiencing any TFA in early and middle adolescence was associated with accessing sexual health services in the last 12 months in unadjusted analyses (RR 1.48; 95% CI 1.11, 1.99) and with IPV victimization (RR 1.63; 95% CI 1.01, 2.65), but these ass

ociations were not statistically significant after adjusting for covariates. In multivariable analysis, participants who had experienced any TFA in early and middle adolescence were more likely to have a teen pregnancy (risk ratio (RR) 1.71; 95% confidence interval (CI) 1.02, 2.84) and any STI diagnosis in emerging adulthood (RR 2.22; 95% CI 1.19, 4.16), adjusting for covariates (Table 4). Any experience of TFA in early or middle adolescence (wave 2–5) was not associated with condom use at last sex, depression, anxiety, IPV perpetration or TFA in emerging adulthood (wave 6). Stratified estimates were similar by sex at birth but confidence intervals were wide because outcome events were small by sex at birth, limiting power (Appendix Table 2). When examining intensity of TFA exposure, those who had medium (RR 2.75; 95% CI 1.08–6.98) and high levels (RR 4.71; 95% CI 1.50–14.8) of TFA exposure at Waves 2–5 had a higher risk of IPV victimization, compared to those with no TFA exposure, adjusting for covariates (Supplementary Table 3).

**Table 4:** Risk ratio (RR) and 95% confidence intervals (Cis) for the association between TFA in early and middle adolescence (wave 2–5) on outcomes in emerging adulthood (wave 6)

	Unadjusted RR (95% CI)	Adjusted RR^a^ (95% CI)
Teen pregnancy (pregnancy < age 20)	**1.96 (1.20**,** 3.22)**	**1.71 (1.02**,** 2.84)**
Accessed sexual health services in last 12 months	**1.48 (1.11**,** 1.99)**	1.27 (0.95, 1.70)
Any STI diagnosis	**2.57 (1.42**,** 4.67)**	**2.22 (1.19**,** 4.16)**
Condom use at last sex	0.88 (0.71, 1.09)	0.86 (0.69, 1.07)
Symptoms of depression	1.22 (0.82, 1.84)	1.16 (0.76, 1.76)
Symptoms of anxiety	1.15 (0.99, 1.33)	1.03 (0.87, 1.23)
IPV victimization	**1.63 (1.01**,** 2.65)**	1.45 (0.88, 2.41)
IPV perpetration	1.36 (0.83, 2.25)	1.20 (0.71, 2.02)
TFA in phase 2	1.36 (0.92, 2.00)	1.09 (0.72, 1.64)

^a^adjusted for sex, neighborhood disorder, food insecurity, mother’s education level, depression at baseline, substance use at baseline

*RR* Risk ratio, *CI* Confidence interval, *STI* Sexually transmitted infection, *IPV* Intimate partner violence, *TFA* Technology facilitated abuse

Bold *p* < 0.05

## Discussion

This study, which represents one of the few longitudinal studies exploring an association between TFA and SRH [6, 22, 27], found that about one-fifth of youth in this predominantly Latine community were experiencing TFA at each study wave (within a 6-month period) and 40% had experienced TFA at any visit during early and middle adolescence. While there was a slight difference in the proportion of female and male youth who experienced TFA at any visit this difference was not statistically significant (*p* > 0.05). Critical risk factors for TFA in adolescence included facing food insecurity, experiencing higher neighborhood disorder, substance use, and experiencing depression. We also found a relationship between TFA exposure and several SRH outcomes, including teen pregnancy, access to SRH services, and self-reported STI diagnosis, as well as IPV victimization. These findings remained significant in adjusted models for teen pregnancy and self-reported STI diagnosis.

Results add to growing literature on TFA, particularly its association with SRH outcomes. Despite a theorized relationship, prior research has predominately looked only at SRH behaviors for associations with TFA, such as self-reported sexual intercourse under the influence of alcohol or drugs [27, 28]. When health outcomes, as opposed to behaviors, have been examined, they typically focus on mental health [29–32]. Established relationships, however, between in-person IPV and SRH, such as susceptibility to HIV acquisition, serve as a model for understanding TFA exposure on SRH [33–35]. These suggest that violence exposure, even of a psychological nature, may erode physiological, psychological, and behavioral pathways to SRH [34–36]. 

It is important to further integrate TFA in SRH research, healthcare provision, and education. Our results, which indicated that those exposed to TFA visited a health clinic at higher rates than those not exposed, suggest that healthcare providers have an important opportunity to identify this form of partner violence and potentially mitigate health-related outcomes. IPV screening in healthcare settings, already a critical tool for detecting violence and linking individuals to care [37], may be adapted to include questions on digital abuse. In addition to health serves, school settings can offer an effective avenue for addressing TFA and promoting healthy relationships [38]. In states like California, where comprehensive sexual education is mandated by the California Healthy Youth Act [39], integrating content on TFA—including digital consent, online harassment, and boundary-setting—into existing curricula can equip adolescents with the knowledge and skills to recognize abusive behaviors and seek support. Schools can also incorporate this content into broader health or life skills programs, ensuring that education on digital and relational safety reaches all students, regardless of their sexual health curriculum exposure. Finally, these findings also underscore the potential utility of advocacy or empowerment-based responses in both sectors, which center survivors’ needs and offer holistic support through safety planning, legal and digital literacy, and access to care [40]. While most advocacy-based interventions have addressed in-person IPV, these approaches should be expanded to address TFA, especially among youth from marginalized communities such as rural Latine adolescents. Together, these intersecting strategies represent critical entry points to reduce the harm associated with TFA and improve adolescent health outcomes.

Violence research often focuses on victimization among women and girls, given globally higher proportions of violence exposure and more severe impacts for this population [41]. Our study, however, showed that a large proportion of males also experience TFA behaviors. This is in line with research with this study population in early adolescence that showed that approximately 20% of participants experienced in-person dating violence at each visit, with levels similar regardless of sex [19]. Other research on TFA has indicated similar findings, although sometimes pointing towards more detrimental impacts among girls [29]. This points to a need for further research to understand differences by sex, as well as a need for comprehensive healthy relationship education to be incorporated as part of sexual education for all youth.

Few studies have yet examined whether youth of different sexual or gender identities are at higher risk [27]. In this study, we were unable to assess differences by sexual identity or gender identity given measurement issues and because our sample was not large enough. Sexual identity (e.g. gay, bisexual etc.) was only assessed at Wave 6. Sexual attraction was assessed in early and middle adolescence with response options of same-sex, opposite-sex or both/either, or neither, but does not always correlate with identity. We also did not have self-reported sexual identity which would influence TFA risk, and sexual attraction changed widely across the study period. In terms of gender identity, gender was not asked at enrollment and few participants at later waves identified as genders other than cisgender men and cisgender woman. Given the limited sample, we could not examine differences for AYA who identified as transgender, nonbinary, or other genders. We will further assess differences in TFA by sexual and gender identity in our ongoing, supplementary work to enroll additional participants who identify as LGBTQ + where we will be able to examine these questions with more power.

Finally, our findings on associated risk and protective factors, particularly those related to food insecurity, neighborhood safety, and substance use suggest further avenues for prevention. There is an established body of evidence pointing towards the effectiveness of economic empowerment interventions (which could reduce food insecurity and housing insecurity, which are often associated with violence exposure), the use of bystander interventions to enhance environmental safety, treatment for substance use, and parenting programs for the prevention of violence [42]. Given the broader structural inequities affecting Latine youth, a health equity framework underscores the need for multi-level interventions that address these social determinants of health and reduce disparities in SRH outcomes [13, 43]. 

There are some limitations to this work. For one, our sample was relatively small (*n* = 386). While this is sufficient for our primary analysis, it makes our stratified analysis by sex, and our analysis by intensity level, less robust. A larger sample may be necessary to make stronger conclusions around the relative impact among youth of different sexes, gender identities, and sexual orientation, and of varying intensities of TFA, which would be important for future programming. Our results also do not provide a causal link between TFA and SRH outcomes. However, the longitudinal nature of our data strengthens the findings, particularly in relation to limited longitudinal data on TFA [27]. Another limitation was our reliance on retrospective self-reported STI measures. Although STI testing data were available, testing was declined by more than one-third of participants. Self-reported data are subject to potential inaccuracies, influenced by social desirability bias, and may not account for asymptomatic or undiagnosed infections. Our use of Computer-Assisted Self-Interviewing (CASI), however, should have minimized reporting biases related to STI diagnosis. Lastly, there were slight differences in sex and coital debut (< age 15) among participants who did and did not re-enroll in wave 6 of the study, but we did not find any differences in TFA. Models adjusted for sex but is possible that estimates may be biased towards the null.

Two further unmeasured concepts, which may influence our understanding of Latine youth vulnerability to TFA are cultural norms and onset of puberty. While we examined immigrant generational status and found no significant differences in TFA exposure across groups, we did not directly assess family customs or cultural traditions. Generation status may not fully reflect important cultural dimensions—such as traditional gender norms, family communication, or acculturation stress—that could shape how youth experience or report TFA [44]. Although these factors have had inconsistent associations with IPV [45], they may be important to explore in future research. Similarly, we did not assess pubertal timing, such as early menarche (before age 11), which is associated with elevated risk for IPV and other SRH concerns (e.g., early sexual activity, depressive symptoms, STIs) [46, 47]. 

## Conclusions

Notwithstanding limitations, this longitudinal study on the association between TFA exposure and SRH outcomes adds important evidence to our understanding of the impact of TFA on SRH for Latine youth. Its identification of critical risk factors such as food insecurity, neighborhood safety, and depression point towards important avenues of intervention, as does the finding that males and females may face similar levels of TFA. While the relationship between TFA and teen pregnancy and self-reported STI diagnosis are concerning, increased healthcare visits among TFA-exposed youth offer further opportunities for the healthcare system to detect and respond to violence using screening and advocacy-based interventions. Future research should explore the pathways between TFA and SRH outcomes, as well as conduct participatory work with youth to design youth-friendly and culturally relevant prevention strategies that can be integrated into existing sex education or life skills school curricula.

## Supplementary Information

Supplementary Material 1.

## Data Availability

Data are not publicly available but can be requested from the corresponding author.

## Abbreviations

ACASI: Audio-computer assisted self-interview
AYA: Adolescents and young adults
Cis: Confidence intervals
GAD: Generalized anxiety disorder
IPV: Intimate partner violence
IQR: Interquartile range
RR: Risk ratio
SRH: Sexual and reproductive health
STIs: Sexually transmitted infections
TFA: Technology-facilitated abuse
US: United States
USDA: United States Food Administration
