# Supplementary material for: Technology-facilitated abuse as a risk factor for STI and pregnancy in early adulthood: evidence from latine adolescents in California
Source: Reprod Health. 2025 Sep 30;22:166. doi: 10.1186/s12978-025-02128-5 (PMC12486503; doi:10.1186/s12978-025-02128-5)
Supplement: Supplementary file 1 — Supplementary Material 1. [file 12978_2025_2128_MOESM1_ESM.docx]

Appendix table 1: Experiences of TFA at wave 2 by sex at birth

|  | |  |  |  |  |  |
| --- | --- | --- | --- | --- | --- | --- |
|  |  |  |  |  |  |  |
|  | **Sex at Birth** | | | | | |
|  | **Male (n=283)** | | **Female (n=316)** | | **Total (n=599)** | |
|  | No. | % | No. | % | No. | % |
| Partner repeatedly contacted you to see where you were/who you were with | | | |  |  |  |
| Never | 43 | 28.9 | 42 | 29.0 | 85 | 28.9 |
| A few times | 50 | 33.6 | 55 | 37.9 | 105 | 35.7 |
| Once or twice a month | 12 | 8.1 | 13 | 9.0 | 25 | 8.5 |
| Once or twice a week | 17 | 11.4 | 10 | 6.9 | 27 | 9.2 |
| Every day or almost every day | 27 | 18.1 | 25 | 17.2 | 52 | 17.7 |
| Partner made mean or hurtful comments to you | | | |  |  |  |
| Never | 121 | 81.2 | 123 | 84.2 | 244 | 82.7 |
| A few times | 23 | 15.4 | 21 | 14.4 | 44 | 14.9 |
| Once or twice a month | 2 | 1.3 | 1 | 0.7 | 3 | 1.0 |
| Once or twice a week | 1 | 0.7 | 0 | 0.0 | 1 | 0.3 |
| Every day or almost every day | 2 | 1.3 | 1 | 0.7 | 3 | 1.0 |
| Partner spread rumors about you | | | |  |  |  |
| Never | 130 | 87.2 | 129 | 88.4 | 259 | 87.8 |
| A few times | 14 | 9.4 | 16 | 11.0 | 30 | 10.2 |
| Once or twice a month | 3 | 2.0 | 0 | 0.0 | 3 | 1.0 |
| Once or twice a week | 0 | 0.0 | 1 | 0.7 | 1 | 0.3 |
| Every day or almost every day | 2 | 1.3 | 0 | 0.0 | 2 | 0.7 |
| Partner made a threatening or aggressive comment to you | | | |  |  |  |
| Never | 140 | 94.0 | 139 | 95.9 | 279 | 94.9 |
| A few times | 6 | 4.0 | 6 | 4.1 | 12 | 4.1 |
| Once or twice a month | 1 | 0.7 | 0 | 0.0 | 1 | 0.3 |
| Once or twice a week | 2 | 1.3 | 0 | 0.0 | 2 | 0.7 |
| Partner tried to get you to talk about sex when you did not want to | | | |  |  |  |
| Never | 132 | 88.6 | 125 | 85.6 | 257 | 87.1 |
| A few times | 10 | 6.7 | 17 | 11.6 | 27 | 9.2 |
| Once or twice a month | 2 | 1.3 | 2 | 1.4 | 4 | 1.4 |
| Once or twice a week | 2 | 1.3 | 1 | 0.7 | 3 | 1.0 |
| Every day or almost every day | 3 | 2.0 | 1 | 0.7 | 4 | 1.4 |
| Partner asked you to do something sexual that you did not want to do | | | |  |  |  |
| Never | 132 | 88.6 | 125 | 86.2 | 257 | 87.4 |
| A few times | 10 | 6.7 | 18 | 12.4 | 28 | 9.5 |
| Once or twice a month | 2 | 1.3 | 0 | 0.0 | 2 | 0.7 |
| Once or twice a week | 1 | 0.7 | 1 | 0.7 | 2 | 0.7 |
| Every day or almost every day | 4 | 2.7 | 1 | 0.7 | 5 | 1.7 |
| Partner posted or publicly shared a nude or seminude picture of you | | | |  |  |  |
| Never | 141 | 94.6 | 142 | 97.9 | 283 | 96.3 |
| A few times | 6 | 4.0 | 3 | 2.1 | 9 | 3.1 |
| Once or twice a month | 0 | 0 | 0 | 0 | 0 | 0 |
| Once or twice a week | 0 | 0 | 0 | 0 | 0 | 0 |
| Every day or almost every day | 2 | 1.3 | 0 | 0.0 | 2 | 0.7 |

Appendix table 2: Risk ratio (RR) and 95% Confidence intervals (Cis) for association between TFA from wave 2-5 with outcomes at wave 6, stratified by sex at birth

|  | **Male Sex at Birth** | **Female Sex at Birth** |
| --- | --- | --- |
|  | **Adjusted RR (95% CI)** | **Adjusted RR (95% CI)** |
| Teen pregnancy (pregnancy < age 20) | **3.87 (1.08, 23.77)** | 1.37 (0.78, 2.40) |
| Accessed sexual health services in last 12 months | 2.72 (1.16, 6.41) | 1.15 (0.85, 1.56) |
| Any STI diagnosis | 5.38 (1.18, 24.68) | 1.78 (0.89, 3.52) |
| Condom use at last sex | 0.86 (0.61, 1.20) | 0.87 (0.65, 1.15) |
| Depression | 1.45 (0.70, 3.04) | 1.04 (0.64, 1.68) |
| Anxiety | 1.06 (0.81, 1.37) | 1.12 (0.93, 1.34) |
| IPV victimization | 1.55 (0.79, 3.08) | 1.35 (0.68, 2.71) |
| IPV perpetration | 1.48 (0.60, 3.63) | 1.05 (0.57, 1.98) |
| TFA at wave 6 | 1.45 (0.67, 2.71) | 0.99 (0.61, 1.62) |

*adjusted for sex, neighborhood disorder, food insecurity, mother’s education level, depression, substance use; Bold p<0.05

Appendix Table 3: Risk ratio (RR) and 95% Confidence intervals (Cis) for the association between TFA intensity category in phase 1 on outcomes at phase 2

|  | **Unadjusted RR (95% CI)** | | | **Adjusted RR (95% CI)** | | |
| --- | --- | --- | --- | --- | --- | --- |
|  | **Low** | **Med** | **High** | **Low** | **Med** | **High** |
| Teen pregnancy (pregnancy < age 20) | 1.02 (0.49-2.13) | 0.93 (0.37-2.37) | 1.52 (0.34-6.81) | 1.03 (0.47-2.24) | 0.99 (0.37-2.6) | 2.26 (0.43-11.80) |
| Accessed sexual health services in last 12 months | 0.69 (0.42-1.13) | 0.72 (0.39-1.35) | 0.59 (0.14-2.46) | 0.71 (0.41-1.20) | 0.87 (0.45-1.69) | 0.53 (0.12-2.41) |
| Any STI diagnosis | 0.79 (0.34-1.87) | 1.3 (0.51-3.29) | 2.79 (0.77-10.12) | 0.67 (0.27-1.68) | 1.21 (0.46-3.22) | 2.17 (0.47-9.93) |
| IPV victimization | 1.71 (0.66-4.45) | **2.75 (1.00-7.57)** | **4.71 (1.17-18.85)** | 1.39 (0.51-3.74) | 2.25 (0.80-6.34 | **4.96 (1.09-22.54)** |

TFA intensity category represents levels of intensity of TFA exposure where scores of less than 3 = low intensity, scores > 3, but less than 10 = medium intensity, and scores > 10 = high intensity. The reference is no exposure to TFA.

*adjusted for sex, neighborhood disorder, food insecurity, mother’s education level, depression at baseline, substance use at baseline

Bold p<0.05
